# Derivation and validation of a preoperative risk model for postoperative mortality (SAMPE model): An approach to care stratification

**DOI:** 10.1371/journal.pone.0187122

**Published:** 2017-10-30

**Authors:** Luciana Cadore Stefani, Claudia De Souza Gutierrez, Stela Maris de Jezus Castro, Rafael Leal Zimmer, Felipe Polgati Diehl, Leonardo Elman Meyer, Wolnei Caumo

**Affiliations:** 1 Department of Surgery, Universidade Federal do Rio Grande do Sul (UFRGS), Porto Alegre, RS, Brazil; 2 Anesthesia and Perioperative Medicine Service, Hospital de Clínicas de Porto Alegre (HCPA), Porto Alegre, RS, Brazil; 3 Medical Science Postgraduation Program,- Universidade Federal do Rio Grande do Sul (UFRGS), Porto Alegre, RS, Brazil; 4 Laboratory of Pain and Neuromodulation, Hospital de Clínicas de Porto Alegre, Porto Alegre, RS, Brazil; 5 Department of Statistic—Instituto de Matemática e Estatística—UFRGS, Porto Alegre, RS, Brazil; George Washington University, UNITED STATES

## Abstract

Ascertaining which patients are at highest risk of poor postoperative outcomes could improve care and enhance safety. This study aimed to construct and validate a propensity index for 30-day postoperative mortality. A retrospective cohort study was conducted at Hospital de Clínicas de Porto Alegre, Brazil, over a period of 3 years. A dataset of 13524 patients was used to develop the model and another dataset of 7254 was used to validate it. The primary outcome was 30-day in-hospital mortality. Overall mortality in the development dataset was 2.31% [n = 311; 95% confidence interval: 2.06–2.56%]. Four variables were significantly associated with outcome: age, ASA class, nature of surgery (urgent/emergency vs elective), and surgical severity (major/intermediate/minor). The index with this set of variables to predict mortality in the validation sample (n = 7253) gave an AUROC = 0.9137, 85.2% sensitivity, and 81.7% specificity. This sensitivity cut-off yielded four classes of death probability: class I, <2%; class II, 2–5%; class III, 5–10%; class IV, >10%. Model application showed that, amongst patients in risk class IV, the odds of death were approximately fivefold higher (odds ratio 5.43, 95% confidence interval: 2.82–10.46) in those admitted to intensive care after a period on the regular ward than in those sent to the intensive care unit directly after surgery. The SAMPE (Anaesthesia and Perioperative Medicine Service) model accurately predicted 30-day postoperative mortality. This model allows identification of high-risk patients and could be used as a practical tool for care stratification and rational postoperative allocation of critical care resources.

## Introduction

Perioperative risk is multifactorial. It depends on the interaction between anaesthetic, surgical, and patient-specific aspects. The perioperative period can be particularly hazardous to patients because it involves several transfers of care [1,2]. Such fragmentation and discontinuity of care might lead to a system-wide fragility that compromises patient safety, especially in high-risk cases[3].To mitigate this, patients at heightened risk of poor outcomes should be as visible as possible; labelling them as such throughout their hospitalization could improve the process and safety of care as a whole, including human resources and technical–administrative aspects. Furthermore, in the context of limited healthcare resources, utilization of critical care resources in the postoperative period is amongst the costliest components of care. This gives rise to several questions: for whom should such specialized care be provided? How can this selection process be made clearer in increasingly crowded and complex health systems?

In recent years, risk management has become a key institutional goal centred on the quality of care, and many surgical risk models and scores have been developed[4]. The ideal stratification tool should be constructed with easily collected preoperative variables that reflect both patient health status and the risk inherent to the surgical procedure. Loss of physiological reserve should also be recognized as a predictor of perioperative vulnerability, and it is essential that the broader characteristics of the patient population of interest be taken into account. The Surgical Risk Scale[5] and de Surgical Mortality Probability model [6] are the proposed indices that come closest to achieving these goals; however, they do not include age as an explanatory variable.

The aim of the present study was to develop a practical approach for stratification of patients undergoing elective or emergent procedures, with satisfactory accuracy, and using feasible, independent preoperative variables. This model would classify patients into risk groups to predict the level of postoperative care required, specifically by making the high-surgical risk group more visible.

## Materials and methods

### Data source and study population

This study was conducted at Hospital de Clínicas de Porto Alegre (HCPA), an 842-bed teaching hospital and referral centre that provides tertiary and quaternary care to patients from across Southern Brazil through the national Unified Health System. Ethical approval for this study was provided by the Ethical Comittee of Postgraduate and Research Group from Hospital de Clínicas de Porto Alegre–Brazil (Chairperson Prof. Eduardo P Passos) on the 13^th^ of June 2014 (CAAE 30776914.1.0000.5327).

Written informed consent was not required, but the authors signed a confidentiality agreement to assess information from institution's database.

We analysed data from all consecutive surgeries performed from January 1, 2012 to December 31, 2013. We first identified 40,505 records from patients who underwent any form of surgery. We excluded those who received only local anaesthesia by the surgeon or whose procedures were diagnostic rather than therapeutic (26,981). Also when more than one surgical procedure was performed during the same hospital admission, only the major procedure was taken into account for analysis. The final study cohort consisted of 13,524 patients. The database included information on patient demographics, functional status (ASA Physical Status classification), nature of surgery (emergency or elective), and degree of surgery (major, intermediate, or minor; detailed definition provided below), as well as postoperative allocation, e.g., regular ward versus intensive care unit. The final outcome during hospitalization was death or survival at hospital discharge. Therefore, the data of the patients who were still in hospital after 30 days or who were discharged before the study period were not followed beyond this point.

### Model development

We used a subsequent approach to select the variables and refine the risk model for surgical mortality. Firstly, only preoperative clinical and surgical routinely available variables with proven accuracy in existing perioperative risk models [5,6] were used. The surgical variables selected were the degree (major, intermediate, or minor) and nature (elective or non-elective) of the procedure. To define surgical severity, we grouped 1200 current terminology codes for similar procedures into subtypes (e.g., bile duct surgery, pulmonary resection). Then, we classified these procedures into major, intermediate, or minor degree, using a categorization scheme based on literature review [6,7] and expert opinions, who considered surgical time, trauma, and predicted bleeding. (S1 Table). The nature of the procedures was categorized as elective or non-elective (urgent and emergency cases).

Variables related to patient physiological reserve included age and ASA Physical Status (ASA-PS) score. As this model was constructed on the basis of institutional data, other clinical predictors, such as cardiac comorbidities, could not be recovered.

A logistic regression model was adjusted to these four independent predictors: two patient-related (ASA-PS, age) and two procedure-related (surgical severity and elective vs non-elective nature). As noted above, death or survival at hospital discharge was the main outcome of interest. Patients were assessed for up to 30 days of hospitalization.

Odds ratio and 95% confidence intervals were calculated to determine the magnitude with which these variables were associated with likelihood of 30-day in-hospital postsurgical deaths. The C-statistic was used to predict the model’s ability to sort patients by outcome. The Hosmer–Lemeshow test was used to check for goodness of fit by comparing the expected and actual deaths in each risk group.

The final model was validated with a new sample (another database from the same institution). The validation dataset was composed of consecutive patients who underwent surgical procedures at the study institution from January to November 2014. The same tests [logistic regression analysis, Hosmer–Lemeshow statistic, receiver operator characteristic (ROC) curve analysis] were applied, using the original sample cut-off point, to confirm the accuracy and calibration of the risk model. All statistical analyses were carried out in the SAS version 9.4.

## Results

### Model development

**Fig 1:**
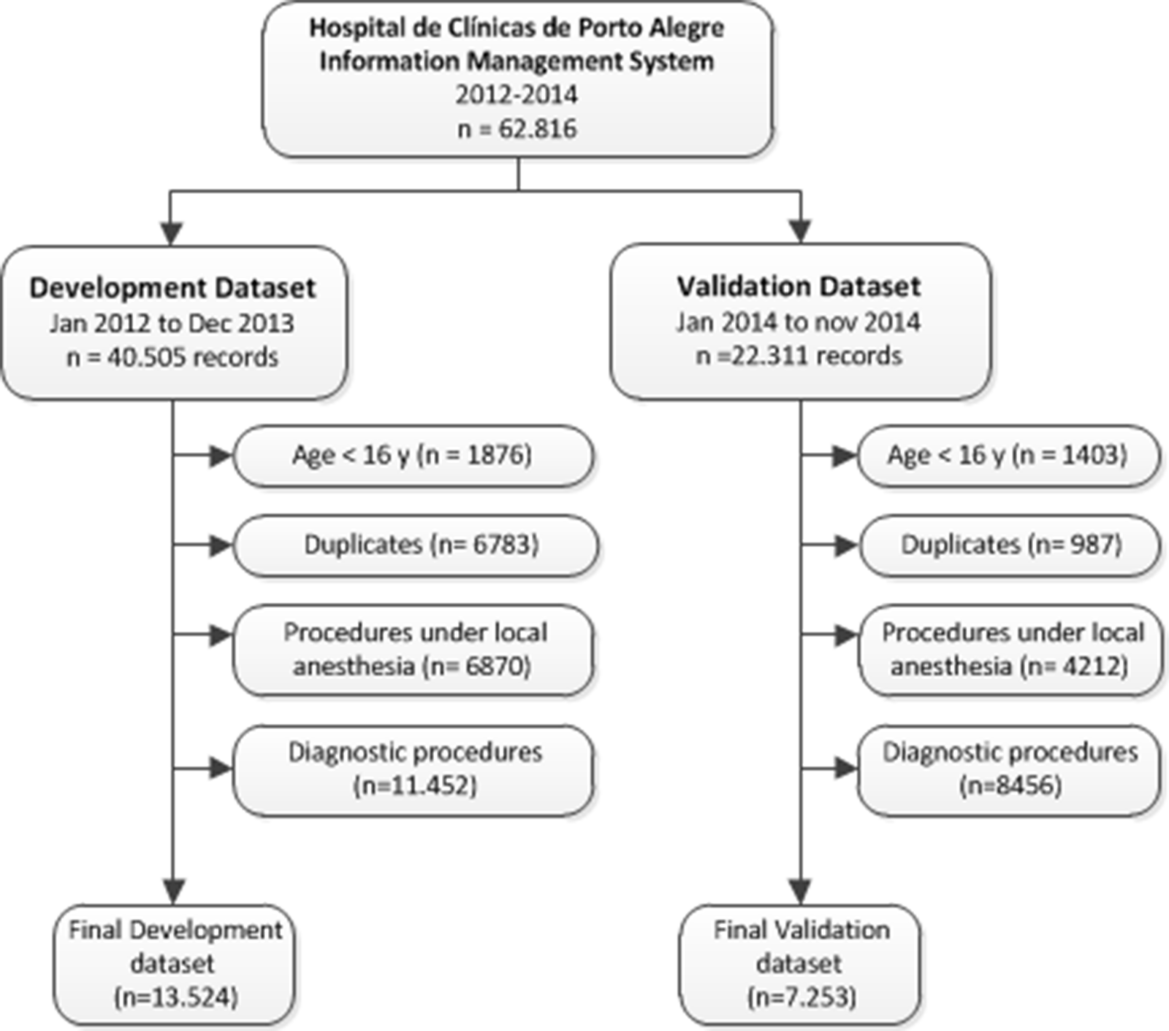
Trial diagram for SAMPE model dataset analysis.

Fig 1 shows the study flow chart. During the 24 months of analysis, 13524 patients comprised the dataset used to develop the model. In this series, there were 311 operative deaths [2.30%; 95% confidence interval (CI): 2.06–2.56%]. Table 1 describes the characteristics of the overall sample and of the 30-day in-hospital postsurgical deaths, stratified by the clinical and surgical variables of interest. The procedures most frequently associated with 30-day in-hospital mortality are listed in S2 Table. Exploratory laparotomy was the procedure most significantly associated with in-hospital postoperative death.

**Table 1:** Characteristics of the overall sample and 30-day in-hospital postsurgical deaths, stratified by clinical and surgical predictors.

	Total sample		Deaths	
	n	Overall %	n	postoperative deaths %
	**13524**	**100**	**311**	**2.30**
**Age**				
15–35	2841	21.00	16	5.14
36–55	4672	34.54	47	15.11
56–75	4901	36.23	161	51.76
>75	1110	8.20	87	27.97
**ASA physical status**				
I	3349	24.76	2	0.64
II	7439	55.00	58	18.64
III	2466	18.23	149	47.90
IV	247	1.82	82	26.36
V	23	0.17	20	6.43
**Nature of procedure**				
Elective	10789	79.77	135	43.40
Urgent	2735	20.22	176	56.59
**Severity of procedure**				
Minor	4809	35.55	50	16.07
Moderate	5593	41.34	66	20.25
Major	3122	23.08	195	62.70

On adjusted logistic regression analysis, the pre-selected variables age, ASA, nature of procedure (elective *vs* non-elective), and procedure degree (major, intermediate, or minor) were found to correlate significantly with the final outcome. Each of these variables contributed to mortality. The probability for mortality is showed by the formula (where Y = 1 if the patient died, *x*_1_ = *age*,*x*_2_ = *ASA*, *x*_3_ = *nature and x*_4_ = *severity*):

$$\begin{array}{l} P(\mathrm{mortality})\;=\;\log\;\frac{P(Y=1|x_{1,}x_{2},x_{3},x_{4})}{1-P(Y=1|x_{1,}x_{2},x_{3},x_{4})}=-10,7506+0,0339\times age+1,7073\times ASA+\\ 1,0672\times nature-0,3699\times intermediate\;severity+0,8966\times major\;severity \end{array}$$

Tests for linearity were performed for ASA status (p = 1.0) and age (p = 0.15) by quartiles test and binned residual plot [8] and it suggested that the linearity supposition was accorded, with increments of 1 year for age and one class for ASA status.

Table 2 lists the variables entered into the model and their respective weights (odds ratios and confidence intervals).

**Table 2:** Variables included in the model with respective odds ratios and confidence intervals.

**Variable**	**Odds ratio**	**95% confidence interval**	**p**
Age	1.035	1.025–1.044	< 0.0001
ASA class	5.514	4.573–6.648	< 0.0001
Surgical severity, intermediate vs minor	0.691	0.467–1.022	0.0641
Surgical severity, major vs minor	2.451	1.750–3.434	< 0.0001
Status, non-elective vs elective	2.907	2.239–3.776	< 0.0001

p-values denote the significance of each variable in improving model predictive capacity (likelihood ratio test).

By analysing these odds ratios with a view to clinical applicability, we drew several conclusions for each of the variables included in the model. Each 1-year increase in patient age was associated with a 1.35-fold increase in the odds of death. Major (vs minor) surgery was associated with a 2.45-fold increase in the odds of death, while each increment in ASA class led to a 5.51-fold increase. Urgent or emergency surgery increased the odds of death by 2.9 compared to elective surgery.

The accuracy of the final logistic regression model was assessed by its discriminant capacity and calibration. The C-statistic for prediction of in-hospital mortality in the derivation cohort was 0.9137, indicating excellent discrimination. The Hosmer–Lemeshow goodness-of-fit statistic of 13.28 (p = 0.125) in the derivation dataset reflects acceptable model calibration.

A sensitivity of 85.2% and specificity of 81.7% were obtained for the adjusted model, considering a cut-off value of 0.02 for the predictive probability of death. Full sensitivity and specificity data are provided in S3 Table.

**Fig 2:**
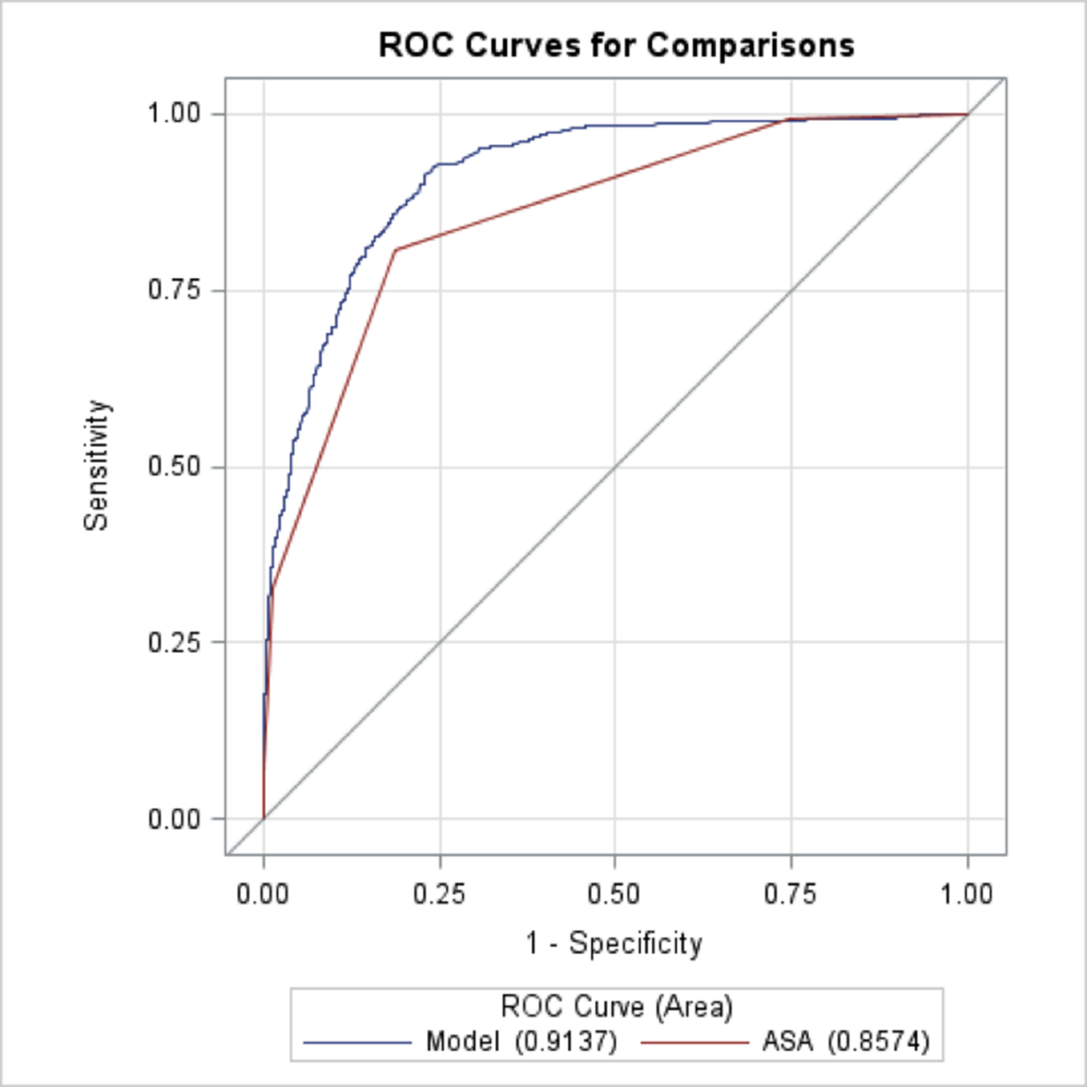
ROC curve calculated using the development SAMPE model dataset compared to the ASA model.

Moreover, the proposed model was compared with a model where the ASA-PS classification was the only predictor, and it added a significant incremental increase in the area under the receiver operating characteristic (AUROC) curve, from 0.857 to 0.913 (p < 0.0001) (Fig 2).

The cut-off sensitivity limit mentioned above yielded four classes of postoperative in hospital all-cause mortality risk:

Class I–probability of death: <2%;
Class II–probability of death: between 2 and 5% (2% ≤ p<5%);
Class III–probability of death: between 5 and 10% (5% ≤ p<10%);
Class IV–probability of death: ≥10%.

Comparisons of the observed and predicted mortality rates for each class (Table 3) were indicative of model consistency and very good calibration, confirming the results of goodness-of-fit testing [9].

**Table 3:** Patient mortality in the derivation cohort, stratified by risk class according to the SAMPE model.

**Risk class** **(Predicted mortality)**	**Total** **(n = 13.524)**	**Deaths (%)** **(n = 311)**
Class I–probability of death: <2%;	10.161	28 (0.28)
Class II–probability of death: between 2 and 5%	1.503	49 (3.26)
Class III–probability of death: between 5 and 10%	915	76 (8.31)
Class IV–probability of death: ≥10%	944	158 (16.74)

### Model validation and utilization

The discriminant ability and calibration of the final model were than assessed in another validation cohort from the same institution, composed of 7253 patients. The high sensitivity (86.4%) and specificity (81.4%) obtained for prediction of in-hospital mortality at a cut-off value of 0.02 confirmed the accuracy of the final model, which we named “SAMPE”, after our institutional affiliation (*Serviço de Anestesia e Medicina Perioperatória*, Anaesthesia and Perioperative Medicine Service). The C statistic for the validation dataset was 0.922. Also, the calculation of the Hosmer–Lemeshow goodness-of-fit statistic for each decile of risk showed a good concordance between observed and predicted deaths at 30 days (x^2^ test = 4.27 –p = 0.89).

**Fig 3:**
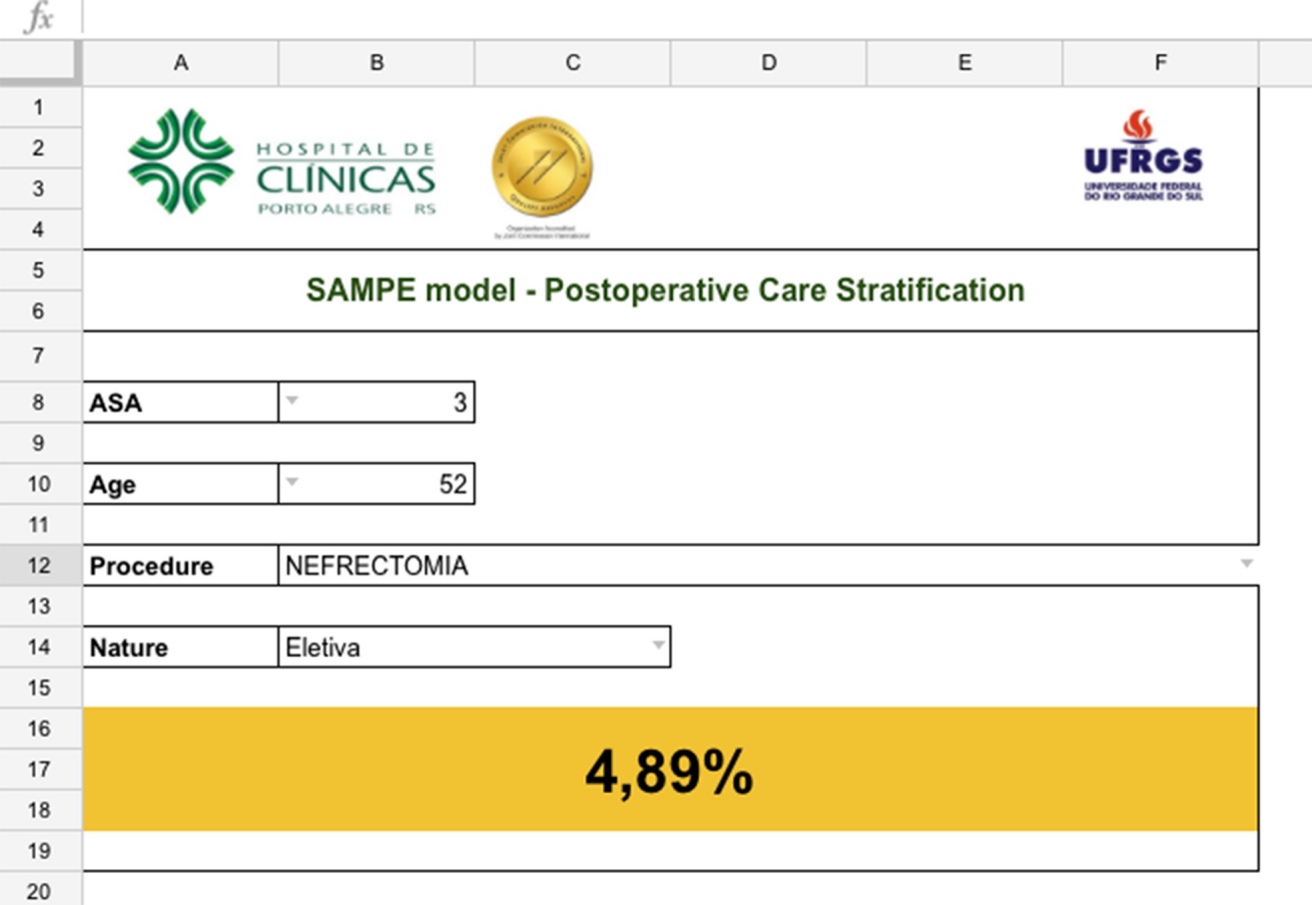
Model calculator developed in the Google Docs platform.

In-hospital death probability was calculated and tabulated for all possible combinations of variables predicted into the model. We also developed an automated on-line table as shown in Fig 3, that calculates the predicted probability of death for each possible combination of variables. This tool will be used to overcome what would otherwise be a considerable challenge—performing a calculation based on a logistic regression equation at the patient’s bedside. The calculator is available at https://www.hcpa.edu.br/downloads/pesquisa/sampe.xlsx.

Although developed as a risk prediction tool before surgery, the SAMPE model, as any other risk model, should ideally be adjusted for use in a new population.

### Worked example for prediction of intensive care unit admission

To illustrate application of the final model, we evaluated postoperative allocation according to SAMPE risk status. The role of intensive care in the management of high-risk surgical patients was analysed. First, surgical admissions to intensive care units were stratified into two groups: patients transferred directly from the operating theatre to the ICU versus patients transferred to ICU after a period of care in the post-anaesthesia recovery room or on a regular ward. A logistic regression model for mortality was done only on the very high risk patients (class IV), considering the predictors used in the original model plus early or late ICU admission (Table 4). Overall, 944 patients were classified as having very high surgical risk (≥10% mortality, i.e., SAMPE class IV). Of these patients, 158 (17%) died. The mortality odds ratio from patients admitted late versus early in ICU was 5.431 (IC 2.820–10.462). The remained patients that died in this category (risk class IV) were not admitted at any time in the intensive care unit (47 patients).

**Table 4:** Mortality-adjusted logistic regression model parameters for high-risk patients (n = 944) and their odds ratio estimates for each predictor.

Predictors	Beta	Standard error	OR	95% CI
**Age**	0.055	0.01	1.057	1.03–1.07
**ASA**	1.757	0.210	5.8	3.83–8.76
**Surgical Severity**				
Low	Ref	-	-	-
Intermediate	-0.177	0.428	0.838	0.36–1.94
Major	0.416	0.367	1.517	0.73–3.11
**Nature**				
Elective	Ref	-	-	-
Urgent/Emergent	1.322	0.295	3.753	2.10–6.69
**ICU admission**				
Early ICU	Ref	-	-	-
No ICU admission	1,454	0.24	0.23	0.14–0.37
Late ICU	3.146	0.327	5.431	2.82–10.46

## Discussion

Statistical risk models for prediction of mortality can be seen as adjuncts to diagnosis, and are best used to enhance perioperative risk reduction strategies. The greatest challenges are to incorporate the chosen model into the care process and ascertain its impact on postoperative outcomes. In this study, we used a dataset of 13524 patients to construct a preoperative model based on clinical and surgical variables, to stratify adult patients into risk classes of in-hospital mortality probability for general surgery. After adjustment and refinement, we validated the model on 7253 patients with a high degree of accuracy. The main strength of our model is that we translate the mathematical model into an automated on-line table that informs the risk and divides it into four categories. This approach creates an efficient risk communication system to the collaborative teams, being its two main goals to predict postoperative complications and to prevent the failure to rescue.

It has already been demonstrated that postoperative death rates oscillate widely across hospitals, even if they have similar complication rates. The hospitals with the best results invest their efforts in the ability of effectively rescue a patient from a complication once it occurs: from timely recognition to effective management[10]. Ferraris et al [11] showed that 20% of patients with the greatest risk for developing postoperative complications account for 90% of failure to rescue. Therefore, identifying these high-risk patients and implementing timely recognition and treatment of early complications are the best opportunity to intervene and limit failure to rescue.

### The model

The high performance of the SAMPE model in the validation cohort (AUROC = 0.913) confirms its consistency. Unfortunately, all risk models currently in use have limitations. Some employ the same variables we selected[5–7] but have limited generalizability and are not easily applied at the bedside. Others, such as the POSSUM score, rely on multiple pre and intraoperative variables and have been shown to overestimate mortality in lower-risk groups [12,13].

One of the strengths of our model is the absence of multiple variables or excessive analyses, which could result in overfitting. Another advantage is its applicability at bedside where it can be used preoperatively, without intraoperative data or laboratory results.

The few clinical and surgical variables selected were powerful predictors of the outcome of interest. Therefore, if a pre-selected combination of variables can explain a phenomenon with the same level of accuracy as a more complex model, the former should be preferred; according to the law of parsimony[14].

It’s known that the models considering surgical and clinical variables have greater accuracy. The comparison of some mortality models using only preoperative variables is shown in Table 5.

**Table 5:** Mortality models with pre-operative variables.

Model	Variables included in the model	Outcome	Population	AUROC (CI)	Comments
**SORT model** [15]	ASA, Surgical Nature, High risk specialty, Surgical Severity, Cancer, Age	Predicted risk of 30-day mortality	General non-cardiac surgery (n = 16.788)	0,91 (0,88–0,94)	It’s a multicenter study in United Kingdom that used a specific surgical severity classification. ROC curve comparing this model with Surgical Risk Scale and ASA was superior. It needs an app web-base calculator.
**Surgical Mortality Probability Model, (SMP-M)**[6]	Surgical severity, ASA, Surgical Nature	30 day mortality	General surgical patients, (n = 298.772)	0.89	It’s a model based on the American College of Surgeons Program database (ACS NSQIP). It exhibited good discrimination compared to the 35-variable ACS NSQIP risk adjustment model.
**mE-PASS** [16]	Age, Severe Pulmonary disease, Severe heart disease, Diabetes mellitus, ASA class, Performance status, Surgical Procedures	In-hospital mortality and 30 day mortality	General surgical patient (n = 5.272)	In hospital mortality: 0.86 (0.79–0.92) 30 day mortality: 0.81 (0.66–0.96)	Model derived from the Japanese National Health Care Reimbursment System. Good accuracy compared to models that included intra-operative variables (E-PASS and POSSUM).
**Lee Cardiac Risk Index** [17]	High risk surgery *(retroperitoneal*, *intrathoracic*, *suprainguinal vascular)*, ischemic heart disease, heart failure, cerebral vascular disease, renal insufficiency, diabetes mellitus	Cardiac mortality up to 30 days	General non-cardiac surgery, (n = 108.593)	0.63	The outcome is focused on cardiovascular mortality. Its simple classification of procedures as high-risk versus not high- risk seems suboptimal.
**Surgical Risk Score**[7]	ASA, surgical severity, surgical nature, age	Inpatient mortality	General surgery, (n = 1.849)	0.88 (0.83–0.93)	It was developed and validated in Italy. Subsequent study evaluating this model found it to be poorly predictive of in-patient mortality [16].
**ASA PS**[7]	ASA	Inpatient mortality	General surgical patient (n = 1.849)	0.81 (0.79–0.82)	ASA grade has been used since 1941. In this cohort, it had good accuracy in predicting mortality even being the only predictor.
**Charlson** [18]	19 clinical conditions	30 day mortality	General surgery (n = 2.167)	0.52	The index is designed to predict 1-year mortality. It does not consider the surgical procedure. In this cohort, the index was the least able to predict mortality.
**Surgical Risk** **Scale**[5]	ASA, surgical severity–*(minor*, *intermediate*, *major*, *major plus*, *complex major)*, surgical nature *(elective*, *scheduled*, *urgent*, *emergency)*	Inpatient mortality	Gastrointestinal, vascular, trauma (n = 1.946)	0.95 (0.93–0.97)	Incorporates specific subclassifications: the CEDOP (Confidential Enquiry into Perioperative Deaths) grade and BUPA (British United Provident Association) classification. Transformed the multivariate regression in a pragmatic score.

This study has several limitations. First, the model reflects mortality risk in the patient population of the study facility, and cannot yet be generalized to other care settings or geographic locations. Second, although it was designed to provide a relatively accurate assessment, 2 of 4 (ASA and surgical severity) variables are subjective measures.

Third, it is limited by the fact that the data were obtained retrospectively; further work is needed to compare the accuracy of the SAMPE model to that of other risk models in a multicentre design. Nevertheless, the numbers at our hospital did not differ greatly from rates reported in developed countries. The overall in-hospital mortality of our sample (2.3%) was comparable to the overall mortality in a 7-day European cohort study[19]. The mortality of patients undergoing high-risk procedures, especially laparotomies, was consistent with that recorded in an ongoing audit project at UK hospitals[20], and the mortality of high-risk patients (8.5%) was similar to that found in a study focused on a similar population [21]. Finally, the outcome in-hospital mortality could bypass the no-less important outcome of perioperative complications, as it is a hard endpoint and postoperative complications are more difficult to define and quantify.

### The variables

Age has been identified as an important predictor of increased risk of postoperative mortality. However, it is not age itself that leads to heightened risk, but rather the decline in bodily functions that comes with ageing [22]. The impact of age-associated decline in five domains is demonstrated in the recently developed Frailty Score[23,24] which has been associated with higher expectancy of adverse hard end-points following surgery, including mortality, functional decline, and cardiac complications.

The most significant variable in the SAMPE model is the physical condition of the patient, determined by the ASA-PS classification. Despite its classic, widespread use and subjective nature, this score was not originally developed for prediction of adverse outcomes; nevertheless, adequate inter-reliability in clinical practice was recently demonstrated[25]. Furthermore, it reflects the global health status of the patient, irrespective of the body systems affected by current pathology. The ASA classification has also been used as the main clinical variable in several recent perioperative risk models, such as the SPM-P model[6] and the Gupta model [26]. The predictive performance of these models exceeds that of traditional risk indices such as the Cardiac Index Revised[17] and the ACS-NSQIP model[27]. In order to reduce the subjectivity of the ASA classification, it’s recommendable the use of the recent update published by the American Society of Anesthesiologists, which encompasses ASA-approved class-specific examples belonging to each class (http://www.asahq.org/resources/clinical-information/asa-physical-status-classification-system).

The risk inherent to the type of procedure performed is also of utmost importance. Elective and less complex procedures had the lowest rates of postoperative mortality, while the worst outcomes were found in patients undergoing major procedures. In our study, only the comparison between major versus minor surgeries was significantly predictive in the model. The classification of procedural severity was adapted from the SPM-P model[6] and corrected for local context after consultation with experts from various surgical specialties and analysis of crude procedure-related mortality, since it depends on several factors related to the whole continuum of perioperative care. The electronic tool contains all procedures previously classified by its severity, which reduces inter-user variability.

Non-elective surgery is a recognized risk factor for perioperative mortality, especially in abdominal procedures[28]. In emergent surgery, there is limited time for data collection and preoperative optimization of comorbid states[29]. Furthermore, the lack of structured care in the crowded and hectic setting of the emergency department certainly contributes to insufficient patient preparation and poor definition of the goals of care. It was recently demonstrated in a large English NHS cohort that structural and procedural aspects such as the number of doctors, nurse staffing, available operating rooms, and critical care beds are important modifying factors related to 30- and 90-day post-emergent surgical mortality[30].

In a worked example, we were able to highlight the ability of our model to guide rational utilization of resources, including postoperative intensive-care allocation, through surgical risk stratification. Despite a higher overall mortality rate (16%) and accounting for over 50% of in-hospital deaths, only 29% of very-high risk (class IV) patients in the cohort were admitted to critical care at any time following surgery (204 early admission vs 68 late admission).

Perhaps most importantly, we found that late admission to the intensive care unit was associated with increased mortality. The very high-risk patients admitted to intensive care after a period of recovery on a regular ward had a 5.43-fold greater risk of postoperative death compared with those admitted to intensive care directly after surgery. Our results confirm the previous findings of higher mortality amongst high-risk patients that were not immediately admitted to a critical care unit after surgery in a large NHS trust [31]. One explanation for these rates could be the lack of availability of critical care resources, since only 4.7% of all risk class patients had immediate postoperative ICU allocation. This contrasts with a European 7-day cohort in which 8% of patients were admitted to the ICU postoperatively [19].

It’s important to emphasize that other determinant variables such as respiratory and haemodynamic instability or trans-operative complications were not included in the pre-operative model. Thus, the model could not be the only source of ICU admission but it may be a useful tool in the allocation decision, especially when there are scarce critical bed resources.

We believe that recognition, identification, and increased visibility of patients with high perioperative risk could make a greater contribution to improving the quality and safety of care than would simply ensuring the availability of critical care resources.

This objective risk assessment could be used to identify which patients must be actively followed in the postoperative period. Multidisciplinary postoperative care teams could also be created, with a view to providing enhanced, patient-centred care and improving postoperative outcomes.

**Fig 4:**
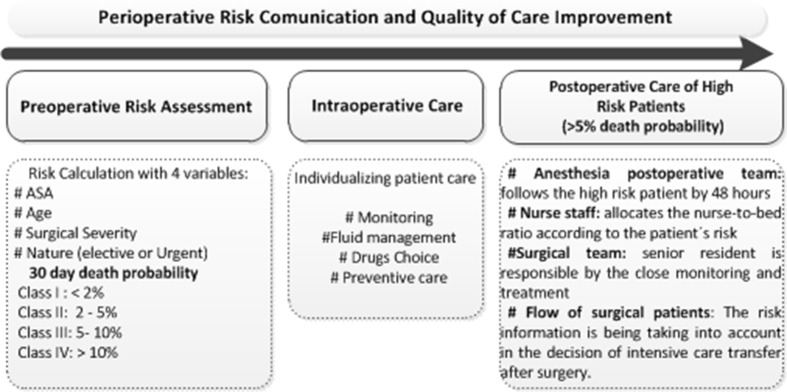
Flow of the high-risk patient’s care.

Accordingly, some processes are been encouraged to be implemented by different caregivers after adopting SAMPE risk model adoption during the high-risk patients’ hospitalization: (i) The postoperative and acute pain team follows them for 48 hours; (ii) the surgeon team assigns senior residents to care for these patients; (iii) the internal medicine co-management is implemented and optimized; (iv) the nurse staff staggers care by prioritizing high-risk patients, evaluating the vital signs more often than usual, and defining nurse-to-bed ratio according to patient’s risk. Additionally, the classification is taken into account in the decision of a possible patient transfer to the ICU after surgery (Fig 4).

## Conclusions

Our perioperative mortality risk model exhibited excellent performance with a small set of easily assessed and sustainable variables. Although the model was well validated internally, prospective validation in external samples is crucial. However, accurate identification of high-risk patients is not enough. The key challenge for clinical translation of our findings, as well as a major avenue for future research, is to incorporate our risk model as a component of care and ascertain its impact on patient outcomes. If successful, this could contribute to improved patient safety and more efficient utilization of perioperative care resources.

## Supporting information

S1 Table Surgical severity criteria developed on the basis of surgical opinion leaders and a literature review, adjusted for crude mortality in the study population. (DOCX)

S2 Table Procedures most frequently associated with mortality in the development dataset. (DOCX)

S3 Table Sensitivity and specificity of the model. (DOCX)
