# Supplementary material for: Derivation and validation of a preoperative risk model for postoperative mortality (SAMPE model): An approach to care stratification
Source: PLoS One. 2017 Oct 30;12(10):e0187122. doi: 10.1371/journal.pone.0187122 (PMC5662221; doi:10.1371/journal.pone.0187122)
Supplement: S1 Table — (DOCX) [file pone.0187122.s001.docx]

**S1 Table.** Surgical severity criteria developed on the basis of surgical opinion leaders and a literature review, adjusted for crude mortality in the study population.

| **Surgical severity** | **Definition** | **Procedures included** |
| --- | --- | --- |
| **Minor** | Minimal or minor risk, independent of clinical conditions or anaesthesia  Minimally to moderately invasive procedure | Minor (laparoscopic) gynaecological procedures  Breast surgery  Minor ENT procedures  Minor oral and maxillofacial procedures  Uncomplicated hernia repair  Appendectomy  Minor genitourinary procedures (nephrostomy, cystoscopy, extracorporeal shock-wave lithotripsy, bladder biopsy)  Thyroidectomy  Laparoscopy  Cosmetic plastic surgeries  Minor orthopaedic procedures (tendon release, small-joint surgery)  Haemorrhoidectomy |
| **Moderate** | Moderately invasive procedure  Potential estimated blood loss 500-1500 ml  Moderate risk to patient independent of clinical conditions or anaesthesia | Endovascular aortic aneurysm repair  Minor orthopaedic and spinal procedures (laminectomy, anterior and posterior cervical arthrodesis, hip or knee arthrotomy, open reduction of jaw fractures).  Cholecystectomy (open or laparoscopic)  Ostomy procedures (gastrostomy, ileostomy, colostomy)  Minor chest procedures (mediastinoscopy, sympathectomy, tracheal prosthesis, video-assisted thoracoscopic surgery)  Oesophageal surgery for benign conditions (pyloromyotomy, oesophagomyotomy, fundoplication)  Splenectomy  Hysterectomy  Minimally invasive or minor neurosurgical procedures (brain biopsy, ventriculoperitoneal shunting, endoscopic intracranial surgery, endoscopic third ventriculostomy)  Percutaneous or laparoscopic genitourinary tract procedures (nephrectomy, adrenalectomy, pyeloplasty, pyelolithotomy, transurethral resection of the prostate or bladder, laparoscopic radical nephroureterectomy)  Pleural surgery (pleurodesis) |
| **Major** | Highly invasive procedure  Potential estimated blood loss greater than 1500 ml  Major to critical risk to patient independent of clinical conditions or anaesthesia | Any heart surgery  Exploratory laparotomy  Colorectal resections/bowel anastomoses (colectomy, rectosigmoidectomy, abdominoperineal resection)  Oesophageal surgery for malignant conditions (oesophagectomy, oesophagogastrectomy) or diaphragmatic hernia repair  Arterial bypass procedures (femorofemoral, aortofemoral, axillofemoral, embolectomy)  Amputations (above/below-elbow, above/below-knee, upper limb, lower limb)  Open aneurysm repair (abdominal, thoracic, or thoracoabdominal aortic aneurysm, surgical repair of aortic dissection)  Hepatic or biliary tract procedures (hepatectomy, biliary tract exploration, biliary-enteric anastomosis)  Pancreatectomy  Major orthopaedic procedures (spinal arthrodesis, revision arthroplasty)  Major chest procedures (lobectomy, pneumonectomy, decortication, exploratory thoracotomy, thymectomy)  Major genitourinary tract procedures (radical cystectomy, nephrectomy, radical prostatectomy, Wertheim-Meigs operation, cystenterostomy),  Manor ENT procedures (laryngectomy, mandibulectomy, tumour resections)  Craniotomy for non-vascular conditions (intracranial tumours, hypophysectomy, spinal tumours)  Vascular microsurgery, drainage of subdural or intracerebral hematoma  Retroperitoneal resections/hemipelvectomy |
