# Supplementary material for: Derivation and validation of a preoperative risk model for postoperative mortality (SAMPE model): An approach to care stratification
Source: PLoS One. 2017 Oct 30;12(10):e0187122. doi: 10.1371/journal.pone.0187122 (PMC5662221; doi:10.1371/journal.pone.0187122)
Supplement: S2 Table — (DOCX) [file pone.0187122.s002.docx]

**S2 Table.** Procedures most frequently associated with mortality in the development dataset

| Procedure | Number of Patients | Deaths | % | % over total number of deaths (n=314) |
| --- | --- | --- | --- | --- |
| Laparotomy | 430 | 94 | 17.73 | 29.84 |
| Arterial bypass | 189 | 22 | 11.64 | 7 |
| Colorectal resection | 337 | 21 | 6.23 | 6.68 |
| Gastric resection | 289 | 15 | 5.19 | 4.77 |
| Amputation (vascular) | 168 | 15 | 8.92 | 4.77 |
| Minor neurosurgery | 56 | 17 | 30.36 | 4.45 |
| Ostomy | 111 | 13 | 11.71 | 4.14 |
| Vascular neurosurgery | 66 | 10 | 15.15 | 3.18 |
| Non-vascular neurosurgery | 150 | 7 | 4.66 | 2.23 |
| Bowel anastomosis | 72 | 7 | 9.72 | 2.23 |
