# Supplementary material for: Derivation and validation of a preoperative risk model for postoperative mortality (SAMPE model): An approach to care stratification
Source: PLoS One. 2017 Oct 30;12(10):e0187122. doi: 10.1371/journal.pone.0187122 (PMC5662221; doi:10.1371/journal.pone.0187122)
Supplement: S3 Table — (DOCX) [file pone.0187122.s003.docx]

**Supplement 3.** Sensitivity and specificity of the model

| **Classification Table** | | | | | | | | | |
| --- | --- | --- | --- | --- | --- | --- | --- | --- | --- |
| **Prob. Level** | **Correct** | | **Incorrect** | | **Percentages** | | | | |
|  | **Event** | **Non-Event** | **Event** | **Non-Event** | **Correct** | **Sensitivity** | **Specificity** | **False POS** | **False NEG** |
| 0.000 | 311 | 0 | 13213 | 0 | 2.3 | 100.0 | 0.0 | 97.7 | . |
| 0.020 | 270 | 10548 | 2665 | 41 | 80.0 | 86.8 | 79.8 | 90.8 | 0.4 |
| 0.040 | 234 | 11555 | 1658 | 77 | 87.2 | 75.2 | 87.5 | 87.6 | 0.7 |
| 0.060 | 198 | 11993 | 1220 | 113 | 90.1 | 63.7 | 90.8 | 86.0 | 0.9 |
| 0.080 | 177 | 12290 | 923 | 134 | 92.2 | 56.9 | 93.0 | 83.9 | 1.1 |
| 0.100 | 151 | 12517 | 696 | 160 | 93.7 | 48.6 | 94.7 | 82.2 | 1.3 |
| 0.120 | 129 | 12693 | 520 | 182 | 94.8 | 41.5 | 96.1 | 80.1 | 1.4 |
| 0.140 | 113 | 12801 | 412 | 198 | 95.5 | 36.3 | 96.9 | 78.5 | 1.5 |
| 0.160 | 100 | 12863 | 350 | 211 | 95.9 | 32.2 | 97.4 | 77.8 | 1.6 |
| 0.180 | 92 | 12933 | 280 | 219 | 96.3 | 29.6 | 97.9 | 75.3 | 1.7 |
| 0.200 | 85 | 12978 | 235 | 226 | 96.6 | 27.3 | 98.2 | 73.4 | 1.7 |
| 0.220 | 80 | 13024 | 189 | 231 | 96.9 | 25.7 | 98.6 | 70.3 | 1.7 |
| 0.240 | 71 | 13060 | 153 | 240 | 97.1 | 22.8 | 98.8 | 68.3 | 1.8 |
| 0.260 | 69 | 13092 | 121 | 242 | 97.3 | 22.2 | 99.1 | 63.7 | 1.8 |
| 0.280 | 57 | 13128 | 85 | 254 | 97.5 | 18.3 | 99.4 | 59.9 | 1.9 |
| 0.300 | 51 | 13146 | 67 | 260 | 97.6 | 16.4 | 99.5 | 56.8 | 1.9 |
| 0.320 | 45 | 13152 | 61 | 266 | 97.6 | 14.5 | 99.5 | 57.5 | 2.0 |
| 0.340 | 38 | 13171 | 42 | 273 | 97.7 | 12.2 | 99.7 | 52.5 | 2.0 |
| 0.360 | 34 | 13177 | 36 | 277 | 97.7 | 10.9 | 99.7 | 51.4 | 2.1 |
| 0.380 | 19 | 13187 | 26 | 292 | 97.6 | 6.1 | 99.8 | 57.8 | 2.2 |
| 0.400 | 17 | 13197 | 16 | 294 | 97.7 | 5.5 | 99.9 | 48.5 | 2.2 |
| 0.420 | 11 | 13204 | 9 | 300 | 97.7 | 3.5 | 99.9 | 45.0 | 2.2 |
| 0.440 | 5 | 13207 | 6 | 306 | 97.7 | 1.6 | 100.0 | 54.5 | 2.3 |
| 0.460 | 3 | 13212 | 1 | 308 | 97.7 | 1.0 | 100.0 | 25.0 | 2.3 |
| 0.480 | 1 | 13213 | 0 | 310 | 97.7 | 0.3 | 100.0 | 0.0 | 2.3 |
| 0.500 | 0 | 13213 | 0 | 311 | 97.7 | 0.0 | 100.0 | . | 2.3 |
